# The healthy diver: A cross-sectional survey to evaluate the health status of recreational scuba diver members of Divers Alert Network (DAN)

**DOI:** 10.1371/journal.pone.0194380

**Published:** 2018-03-22

**Authors:** Shabbar I. Ranapurwala, Kristen L. Kucera, Petar J. Denoble

**Affiliations:** 1 Department of Epidemiology, University of North Carolina at Chapel Hill, NC, United States of America; 2 Department of Exercise and Sports Sciences, University of North Carolina at Chapel Hill, NC, United States of America; 3 Divers Alert Network, Durham, NC, United States of America; Uniklinik Koln, GERMANY

## Abstract

**Background:**

Scuba diver fitness is paramount to confront environmental stressors of diving. However, the diving population is aging and the increasing prevalence of diseases may be a concern for diver fitness.

**Purpose:**

The purpose of this study is to assess the demographics, lifestyle factors, disease prevalence, and healthcare access and utilization of Divers Alert Network (DAN) members and compare them with those from the general population.

**Methods:**

DAN membership health survey (DMHS) was administered online in 2011 to DAN members in the United States (US). Health status of DMHS respondents was compared with the general US population data from the Center for Disease Control and Prevention’s Behavioral Risk Factor Surveillance System using two-sided student’s *t*-tests and Mantel-Haenszel chi-square tests. Univariate and multivariate logistic regression analyses were conducted to identify factors associated with healthcare utilization among the DMHS participants.

**Results:**

Compared to the general US population, the DMHS population had lower prevalence of asthma, heart attack, angina, stroke, diabetes, hypertension, hypercholesterolemia, and disabilities (p<0.01); more heavy alcohol drinkers, and fewer smokers (p<0.01); and greater access and utilization (routine checkup) of healthcare (p<0.01). Healthcare utilization in males was lower than among females. Increasing age and increase in the number of chronic illnesses were associated with increased healthcare utilization.

**Conclusions:**

DAN members are healthier than the general US population. DAN members also have better access to healthcare and utilize healthcare for preventive purposes more often than the general population. DAN members appear to have a better fitness level than their non-diving peers.

## Introduction

Recreational sports provide increased physical activity and are associated with better long-term physical and mental health [1,2]. Recreational scuba diving, too, provides these benefits as it tests the physical and mental capabilities of divers as they interact with a set of complex equipment and hostile environment. Scuba diving is a popular sport with estimated 2.4 million scuba divers in the United States in 2016 [3]. Recent studies from Divers Alert Network (DAN), recorded self-reported injury rate of 3 per 100 dives from survey of recreational divers [4], and a diving-related fatality rate of 16.4/100,000 person-years using insurance claims information from insured DAN members [5]. Hence, it is important that divers are at their best both physically and mentally to avoid adverse outcomes like injuries or death. Good physical health helps the diver to cope with the effects of increased ambient pressure and exertion and good mental health helps to remain focused and make quick decisions.

However, the recreational diving population is aging [5,6] and with the increase in age the prevalence of diagnosed and undiagnosed chronic illnesses and cardiovascular disorders among divers is suspected to be increasing [6]. Diving with cardiovascular disorders, disabilities, and other chronic illnesses like diabetes, asthma, etc. may increase a diver’s risk of injuries or death [7–9]. A study comparing cardiac autopsy findings of diving-related fatalities to motor-vehicle fatalities suggested that diving-related fatalities had higher heart mass and left ventricular wall thickness than motor-vehicle fatalities highlighting the role of cardiovascular disorders in diving fatalities [8].

In order to minimize risk due to pre-existing conditions, Australia, France, and United Kingdom mandate annual health exam for recreational divers and require clearance from a dive medicine physician in case of potentially limiting health conditions [10,11]. Others including the United States (US) do not mandate such health exams for recreational divers [10,11]. The diving industry in the US is mainly self-regulated. Training agencies set the minimum age limits for training however there is no upper age limit. Dive operators and instructors screen divers before diving using the World Recreational Scuba Training Council (RSTC) medical statement form [12,13]. In case diver checks any of listed medical issues, a clearance by physician is required [13]. The South Pacific Underwater Medical Society (SPUMS) recommends medical physicals for fitness-to-dive at 45 years of age and every five years thereafter or earlier depending on health status [14]. However, mandatory annual health exam for divers is debated time and again in the US. Consequently, annual health exams are not mandated for divers in the US. Examining the health status of diving population and their healthcare access and utilization will allow us to understand any potential need for mandated health exams.

However, there are no well-defined recreational diving populations in the US. Two previous studies reported health status of US Navy divers, mostly males, who conducted navy dives between 1960–1990 [15,16]. However, these may not be generalizable to the US recreational divers because navy divers conduct more extreme dives, under targeted missions. Besides, with advancement in diving gear and training, recreational diving population in the US today may differ significantly from 30–50 years ago [3]. Other diver health status studies have been conducted in recreational Australian [17,18] and professional French[19] divers but all these studies suffered due to small sample sizes and are not generalizable to US recreational divers [15–19]. The recreational divers from Australia were also much younger than the current diving population, and may not represent the true health status of the current recreational diving population [17,18].

We conducted a health survey among recreational scuba divers who are members of Divers Alert Network (DAN). DAN is a not-for-profit scuba diving membership organization that provides medical assistance, and education and training to divers, monitors diving injuries, and conducts dive medicine research. Surveying DAN recreational scuba diver membership population may provide information about the healthcare needs of one of the largest and well-defined US based recreational scuba diver population. This study describes demographic factors, lifestyle factors, major chronic illnesses, and access to and utilization of health care among the respondents of DAN membership health survey, and compares the health status of DAN’s recreational divers to the health status of the general US population. Additionally, we examine factors associated with healthcare utilization among DAN survey participants.

## Methods

This is a cross sectional study that used DAN membership health survey (DMHS) data to describe the demographic, lifestyle, and health-related factors of DAN membership population. DAN membership health survey was a self-reported online survey conducted from June 1, 2011 to September 30, 2011 among 30,000 randomly selected DAN members. The methods of survey dissemination and data collection have been described previously [4]. Comparison data for the US population were obtained from the 2011 Behavioral Risk Factor Surveillance System (BRFSS) data [20]; these data are weighted for the general US population. The BRFSS is a system of telephonic surveys conducted by the Centers for Disease Control and Prevention each year in all 50 US states among US residents to assess the nation’s health. To ensure comparability to BRFSS results, we used the same survey questions in DMHS. This study was initially approved by the Divers Alert Network institutional review board (# 001–11) on 08 February 2011 and has been annually re-approved since.

### Variables

We identified four groups of variables 1) demographics, 2) lifestyle factors, 3) chronic health conditions, and 4) healthcare access and utilization. Demographics factors such as age was categorized as 18–24 years, followed by 10 year increments as 25–34, 35–44, …, 75 years or more; sex was a binary variable (male/ female); race was categorized as White and non-White; education was categorized as less than high school, high school, some college, or college graduate; annual income was categorizes in $25,000 increments as <25K, 25-49K, > = 50K per year; BMI was categorized as <25 = Normal or underweight, 25 to <30 = overweight, ≥30 = obese. Lifestyle factors such as alcohol use was categorized based on daily average drinking limits, which is 2 drinks per day for men and 1 drink per day for women. Those who drink more than this are considered heavy drinkers [21]. Those who identified themselves as active cigarette smokers were considered “current tobacco smokers” as opposed to those who used to smoke previously or have never smoked cigarettes (current non-smokers). The participants who reported engaging in physical activities or exercises in addition to their regular job were considered physically active, as opposed to physically not active. All chronic health condition related questions were asked as, “Have you been ever told you had…?” Those answering ‘yes,’ were considered prevalent cases of the respective diseases. Healthcare access and utilization was defined as a routine check-up, and the number of primary care providers. A routine check-up was categorized as that occurring within the past year, the past two years, or more than past two years. Primary care provider information was categorized as a binary variable (no providers vs one or more providers).

The DAN and the US populations were compared for demographic factors, lifestyle factors, chronic health conditions, and healthcare access and utilization.

### Statistical methods

Frequency distributions of all the demographic, lifestyle, and chronic health conditions were computed. A two-sampled t-test was used to compare the mean ages among the DAN membership population and the weighted general US population. All other demographic factors, lifestyle characteristics, chronic health conditions, and access to healthcare were compared between the two populations using chi-square analysis.

The predictors of past year visit to physician for routine check-up in the DAN membership population were examined using multivariate logistic regression analysis. The best fitting logistic regression model was identified using akaike information criterion (AIC–lower the better), which included age, number of chronic health conditions (0, 1–2, or 3+), sex, and the binary annual income variable (<$50,000 per year / ≥$50,000 per year), such that:

$$\frac{p(y)}{1+p(y)}=\propto +\beta 1*age+\beta 2*sex+\beta 3*chronic\;health\;conditions+\beta 4*annual\;income$$

Where,

y = visit to a physician for a routine checkup within past one year (binary–yes or no);
p(y) = probability that a DAN member visited a physician within the past one year of taking the DMHS;
p(y) / 1+p(y) = odds of a DAN member visiting a physician within the past one year of taking the DMHS;
Age = categorical, where 18–24 years was the referent group;
Sex = categorical, where females were the referent group;
Chronic health conditions = categorical, where ‘0’ was the referent category; the chronic health conditions considered for this variable were diabetes, heart attack (myocardial infarction), angina, stroke, cardiac pacemaker, history of cardiac surgery, hypercholesterolemia, hypertension, obesity, being a current smoker, and being a heavy drinker;
Annual income = categorical, where participants with less than $50,000 annual income were the referent group;
Odds ratios and 95% confidence intervals (95% CI) of factors associated with DMHS respondent healthcare utilization are reported. All analyses were conducted in SAS 9.4, SAS Inc.

## Results

Of the 30,000 invited DAN members (median age 49 years; 74.1% males), 5,514 responded (18.4% response) to the online survey, and 4,859 (16.2%) submitted all 20 sections of the survey. All analyses were conducted on the 4,859 submitted responses.

### Demographics

In 2011, the median age of DMHS participants who completed the survey was 52 years (range 18–90 years), 73.5% participants were males (n = 3,388; n = 252 missing gender information), 91.7% were White (n = 4,458), 69.5% had a college degree or higher (n = 3,375), 5.1% were unemployed (n = 249), and 73.8% participants had an annual income of more than $50,000 (n = 3,587), while 14.7% (n = 715) did not report their annual income (Table 1). Sex distribution of DMHS respondents was representative of the DAN membership, however, the DMHS respondents were slightly older than the DAN membership population [4].

**Table 1:** Comparison of demographics and lifestyle factors between DAN survey respondents and the general US population (BRFSS data).

Demographic factors	2011		Difference between DAN & US
	DAN	US	
**Age**^**a**^ - mean (median)	50.2 (52)	46.4 (45)	3.8 (7)*
	**(%)**	**(%)**	**(%)**
**Males**	73.5	48.7	24.8*
**White race**	91.7	68.9	22.8*
**Unemployed**	5.1	8.6	-3.5*
**Education**			
< High School	0.4	10.2	-9.8*
High School	4.4	27.7	-23.3*
Some College	22.1	26.1	-4*
College Graduates	69.5	35.9	33.6*
Missing	3.6	-	-
**Income (US$)**			
< 25 K/ year	2.9	26.8	-23.9*
25-49K/ year	8.6	24.2	-15.6*
> = 50K/ year	73.8	49.1	24.7*
Did not report	14.7	-	-
**BMI**			
Overweight	42.1	35.8	6.3*
Obese	20.6	27.4	-6.8*
Overweight + Obese	62.7	63.2	-0.5
**Lifestyle factors**			
**Heavy alcohol drinkers**			
Male	11.5	7.6	3.9*
Female	16.5	5.6	10.9*
**Current tobacco smokers**	5.3	20.1	-14.8*
**Physically active**^**b**^	93.7	72.5	21.2*

Abbreviations: BRFSS–behavioral risk factor surveillance system; DAN–Divers Alert Network

a—age range 18–90 years

b—during 30 days before taking the survey and other than work-related

*p-value < 0.01

The data from the BRFSS suggests that as compared to the DMHS participants, the 18–90 year old general US population in 2011 was younger–median age 45 years (range 7–99 years), *p-value*<0.01; had fewer males (48.7%), *p-value*<0.01; had fewer individuals who identify themselves as White (68.9%), *p-value*<0.01; had fewer people who graduated from college (35.9%), *p-value*<0.01; had more unemployed people (8.6%), *p-value*<0.01; and had fewer people with annual income greater than $50,000 (49.1%), *p-value*<0.01 (Table 1).

In 2011, 20.6% of DHMS participants were obese (BMI > 30) as compared to 27.4% of general US population, *p-value*<0.01. However, when overweight (25 ≤ BMI < 30) and obese were combined, there was no difference between the DHMS participants (63.3%) and the general US population (62.8%), *p-value* = 0.48 (Table 1).

### Lifestyle factors

About 37% of DHMS participants (n = 1784) indicated that they have smoked more than 100 cigarettes in their lifetime, while 5.3% participants were current smokers (n = 257). In comparison, 20.1% of the general US population identified themselves as current smokers in 2011 (*p-value* < 0.01). Among the current smokers in DMHS participants, 55.6% of the current smokers (n = 143) smoke everyday while, 44.4% (n = 114) smoke on some days. Of all the participants 4.1% (n = 200) tried to quit smoking during the 12 months before taking the DMHS (Table 1).

Among DMHS participants 11.5% males (n = 391) and 16.5% females (n = 201) were heavy drinkers compared to 7.6% males, *p-value* < 0.01, and 5.6% females, *p-value* < 0.01, in the general population (Table 1).

Among the DMHS participants, 93.7% did some physical activity other than work-related physical activity in the past 30 days, as compared to 72.5% BRFSS 2011 participants, *p-value* < 0.01 (Table 1).

### Chronic health conditions

The prevalence of hypertension (high blood pressure) in the DMHS participants in 2011 was 24.6% (n = 1196) compared to 31.6% in the US population, *p-value*<0.01. Among the DMHS participants in 2011, the prevalence of hypercholesterolemia (high blood cholesterol) was 35.4% (n = 1718) compared to 38.5% in the US population, *p-value*<0.01 (Table 2).

**Table 2:** Comparison of prevalence of diseases and healthcare utilization between DAN survey respondents and the general US population (BRFSS data).

Diseases	2011		Difference between DAN & US (%)
	DAN (%)	US (%)	
**Asthma**	4.3	8.6	-4.3*
**Angina**	2.9	4.3	-1.4*
**Stroke**	0.8	2.9	-2.1*
**Heart Attack**	1.5	4.3	-2.8*
**Diabetes**	4.0	9.8	-5.8*
**Disability**^**a**^	2.2	7.3	-5.1*
**Hypertension**	24.6	31.6	-7.0*
**Hypercholesterolemia**	35.4	38.5	-3.1*
**Healthcare access/ utilization**			
**Routine check-up**			
Past year	70.3	66.1	4.2*
Past 2 years	15.2	13.7	1.5*
More than 2 years^b^	14.5	20.2	-5.7*
**Primary care provider**			
One or more	84.1	77.6	6.5*
None	15.9	22.4	-6.5*

Abbreviations: BRFSS–behavioral risk factor surveillance system; DAN–Divers Alert Network

a–disability requiring special equipment

b–includes don’t know, not sure.

*p-value < 0.01

The prevalence of major chronic health conditions among the DMHS participants (Table 2), in 2011, was: asthma– 4.3% (n = 207), angina– 2.9% (n = 142), heart attack– 1.5% (n = 72), stroke– 0.8% (n = 36), diabetes– 4.0% (n = 194), and participants who used special equipment for their disability– 2.2% (n = 108). Compared to the DMHS participants, the BRFSS data suggests (Table 2) that in 2011 the general US population had a higher prevalence of asthma (8.6%, *p-value*<0.01), angina (4.3%, *p-value*<0.01), heart attack (4.3%, *p-value*<0.01), stroke (2.9%, *p-value*<0.01), diabetes (9.8%, *p-value*<0.01), and disable people who use some equipment for their disability (7.3%, *p-value*<0.01).

### Access to healthcare and utilization

In 2011, 70.3% DMHS participants (n = 3418) had visited a primary care provider for a regular check-up within the past year (Table 2). In comparison, 66.1% of general US population had visited a primary care provider for a regular check-up within the past year (*p-value* < 0.01). The utilization of healthcare in the DMHS respondents was 85.5% during the past two years, which was higher than the US population (79.8%, *p-value*<0.01) during the same time period. Additionally, 84.1% DMHS respondents said that had a primary care provider, as compared to the US population (77.6%, *p-value*<0.01) (Table 2).

**Fig 1:**
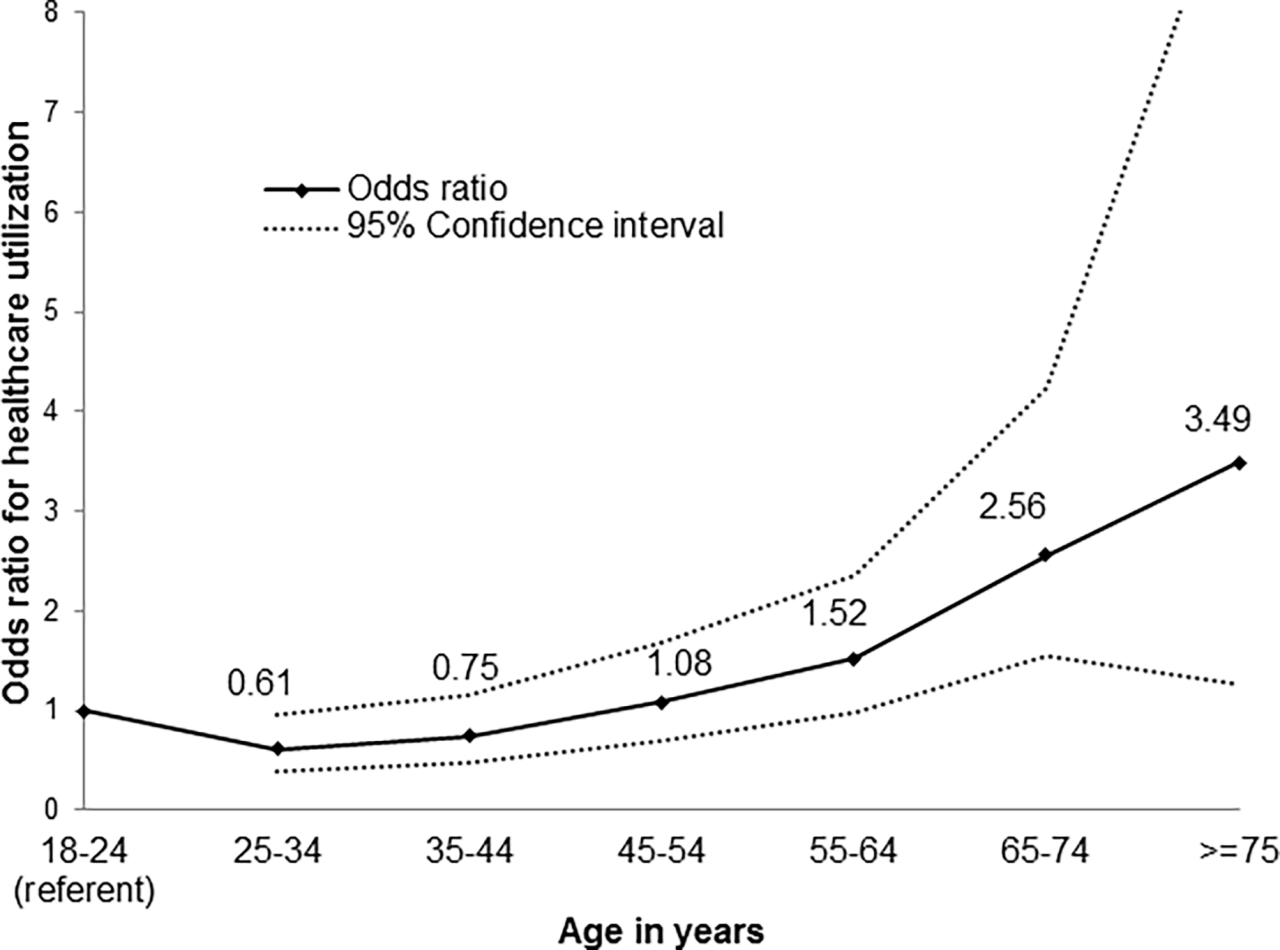
Adjusted odds ratio and 95% CI for healthcare utilization in the past year by age, adjusted for sex, income and chronic health conditions.

The factors associated with healthcare utilization (regular health check-up visit in the past year) are age, sex, the number of chronic disease indicators, and annual income (Table 3). Males had 38% less odds of utilizing healthcare in the past year as compared to females, odds ratio (OR) = 0.62 (95% CI: 0.53, 0.73). The odds of having utilized healthcare for a routine check-up during the past year was lowest for 25–34 years age group, after which they increased with increasing age (Fig 1). Almost two-third of the DMHS participants (n = 3184) reported one or more chronic health condition (range 0–9), and one participant reported nine chronic health conditions. With the addition of every additional chronic health condition, the odds of having visited a physician in the past year for a regular check-up increased by 29%, OR = 1.29 (95% CI: 1.20, 1.37). As compared to those who had an annual income of less than $50,000, those who had an annual income of $50,000 or more had 40% higher odds of visiting a physician in the previous year, while those who did not report their income had 56% higher odds of visiting a physician (Table 3).

**Table 3:** Factors affecting healthcare utilization among DAN survey participants.

	Odds ratio (95% CI)	
	Univariate	Multivariate
**Age (ref = 18–24 years)**		
25–34 years	0.65 (0.42–1.01)	0.61 (0.39–0.95)
35–44 years	0.87 (0.57–1.33)	0.75 (0.48–1.16)
45–54 years	1.36 (0.90–2.06)	1.08 (0.70–1.67)
55–64 years	1.97 (1.30–2.98)	1.52 (0.99–2.36)
65–74 years	3.55 (2.20–5.73)	2.56 (1.55–4.24)
> = 75 years	4.97 (1.82–13.52)	3.49 (1.26–9.65)
**Male (ref = female)**	0.80 (0.69–0.93)	0.61 (0.52–0.72)
**Each additional chronic illness**	1.38 (1.29–1.46)	1.29 (1.21–1.38)
**Income (ref = <$50,000/year)**		
= >$50,000/ year	1.54 (1.28–1.87)	1.40 (1.13–1.72)
Did not report income	1.81 (1.39–2.35)	1.56 (1.18, 2.06)

Abbreviations: CI–confidence intervals; ref–referent.

## Discussion

In this study we observed that as compared to the general US population, the DMHS respondents in 2011 were healthier, wealthier, more educated, more physically active, employed, older, had more males, and had more white participants. However, the DMHS participants included more heavy alcohol drinkers as compared to the general US population. But the DMHS respondents reported greater access to and utilization of healthcare services compared to the general US population. These factors suggest that the DMHS respondents may adhere to general fitness-to-dive recommendations. This is especially evident in that the odds of visiting a provider for a regular checkup in the previous year increased steadily after age 45, which reflects SPUMS recommendations [14]. Additionally, with the increase of every additional chronic health condition the odds of visiting a provider for a regular checkup in the previous year increased by 29%. These findings may suggest that DAN members may already be utilizing healthcare services adequately.

One may question, are DAN members healthier than the general population because they dive? Or is it because the less healthy divers simply drop out? Either of these phenomenon can be explained by the health survivor effect observed among athletes and heavy industry workers, which simply means that only the healthy carry on and hence are the only who can be observed in research studies. It has been noted that athletes and workers in heavy industry or farm workers are usually healthier as compared to the general population because the unhealthy among them drop out [22–24]. Actively diving DAN members^2^ are a special population that may display a similar effect. The fact that DMHS respondents are involved in more physical activity and are healthier despite of being older than the general population, further supports the potential of the ‘healthy diver effect.’

Healthcare utilization was lower among 25–44 years age group as compared to 18–24 year olds. This may be because at the time of the survey in 2011 many 18–24 year olds in the US could have been covered by their parent’s healthcare insurance [25]. Health insurance coverage is associated with healthcare utilization [26]. On the other hand, the 25–44 year olds generally are covered by their employer, spouse, or buy their own insurance and it is possible that some may not have been covered. Greater healthcare utilization among those older than 44 years may be explained due to aging, increasing burden of chronic illnesses, as well as an effect of recommendations by authorities like DAN, RSTC, SPUMS, and Undersea and Hyperbaric Medicine Society [13,14].

It is common knowledge that scuba diving in the United States attracts more males than women, this is also noted by previous studies [17–19], however, the predominance of divers who identify themselves as white has not been noted before. The involvement of specialized gear and their high costs makes scuba diving an expensive sport as compared to other recreational sports. This explains why more respondents of this survey belonged to the higher income category–because they can afford it. Furthermore, higher income is associated with higher education [27,28], all of which explain the high healthcare access and utilization. Higher income, healthcare access, and education may be the reasons why we see higher physical activity, lower obesity, and lower prevalence of chronic disease in the DMHS respondents as compared to the general US population. Overall, this study suggests that the DAN member population is healthier than the general US population.

Other studies have reported health status of other diving populations, too. However, results from previous studies are based on considerably smaller sample sizes than this study (Table 4) [15–19]. Some studies have used restricted data from young divers [13,15,17], or exclusively male US Navy divers [15,16], or new divers [15]. Most previous studies lacked complete health status, lifestyle, or demographic information (Table 4). Previous studies reported considerably lower prevalence of cardiac illnesses, diabetes, obesity, and hypertension compared to our study, which may be attributed to younger population samples and small sample sizes in those studies [15, 17–19]. DMHS respondents also had lower prevalence of smoking as compared to the other two US studies [15,16]. Especially, Dembert et al. noted that 64% of US Navy divers smoked in 1972–1977 [15], which reduced to 7.9% during Chung et al.’s observation [16]–this big decline may be due to changing medical information and awareness about the harmful effects of tobacco [15–18]. The even lower prevalence of smoking among DAN members (lower than the general US population) may represent similar phenomenon to athletes and farm workers who smoke less as compared to the general population, that is smoking may affect their respiratory health which not allow them to participate in their sport or work [22–24]. The prevalence of heavy alcohol drinking among Australian divers was 23.7%, however, the authors of that study defined heavy alcohol consumption at >10 drinks per week for men and women. In contrast, our definition was based on the one the US Centers for Disease Control and Prevention uses, which is 2 drinks/day for men and 1 drink/day for women. The difference in these definitions and the fact that the Australian survey, with a small sample size, was conducted 11 years earlier than ours may explain the differences between that study and ours. However, DMHS respondents did have greater alcohol consumption than the US population, which may be explained by higher socioeconomic status among the DMHS respondents as indicated by higher income and employment.

**Table 4:** Comparison of DAN survey respondent health status with previous reports of health status among other diver populations.

	Recreational			Other diver populations		
	DMHS 2011	Cresp 2000	Taylor 2002	Chung 2011	Dembert 1983	Pougnet 2012
**Population**	DAN (US) Members	Western Australia	Australia	US Navy	US Navy	French Pro-divers
**Data collection**						
Survey method	Online	Mailed	Mailed	Mailed	In-person	Doctor
Data type	Self-report	Self-report	Self-report	Self-report	Self-report	MR
Year	2011	1998–99	2000	2010	1972–77	-
Sample size	4859	515	346	1490	197	200
Response rate	16.2%	54.7%	Not known	27.5%	Volunteers	All patients
**Age**^**a**^**- mean (median)**	50.2 (52)	27 (24)	31–60 = 74%^j^	55.6 (55)	28.3 (-)	37.8 (38)
	**(%)**	**(%)**	**(%)**	**(%)**	**(%)**	**(%)**
**Males**	73.5	72	73.4	99	100	86
**College Graduates**	69.5	-	59.6	51	-	-
**Unemployed**	5.1	-	6.9	3.6	-	-
**Heavy alcohol drinkers**						
Male	11.5	-	23.7^b^	13	>50	-
Female	16.5	-		-	-	-
**Tobacco smokers**^**c**^	5.3	11.6	11.3	7.9	64	29
**Physically active**	94.7	88	-	83.8	-	69.5
**Obese**^**d**^	20.6	4.9	12.7	-	28.9	20.5
**Overweight + Obese**^**d**^	63.3	24	46.8	-	-	40
**Asthma**	4.3	9.7	7.8	8.4^e^	-	-
**Angina**	3.1	-	0.9	10^f^	-	-
**Stroke**	0.8	-	-	1.6	-	-
**Heart Attack**	1.5	0.2^g^	0.3	-	-	1.5^h^
**Diabetes**	4.1	0.2	0.3	7.2	-	0.5
**Disability**	2.2	-	-	59.9^i^	-	-
**Hypertension**	25.1	4.2	3.5	40.8	-	16.5
**High Cholesterol**	36.1	-	-	-	-	50

Abbreviations: DAN–Divers Alert Network; DMHS–DAN members health survey; MR–Medical reports; Pro-divers–Professional divers; a—age in years; b—more than 10 drinks per week, male and female combined; c—current smokers; d—based on BMI distribution; e—asthma + chronic obstructive pulmonary disease; f—angina + heart attack; g—ischemic heart disease; h—cardiac history; i—on disability; j—Taylor 2002 did not report mean and median ages, but reported that 74% participants were in age range 31–60 years.

The DAN membership population is a large well defined population that may represent the general diving population better than the hitherto published data, and the DMHS survey questionnaire was taken from the BRFSS questionnaire which is a well validated survey instrument. However, our study has several limitations that should be considered. First, like all surveys, our results depend on self-reported data. Self-reported surveys could introduce recall bias in our study. However, we used standardized survey questions used in the BRFSS that allowed us valid comparisons with the rest of the US population. This means that if there was a recall bias that would be similar in both the DMHS survey (representing DAN divers) and BRFSS (representing general US population), thereby the difference estimates between these two populations may still be valid. Secondly, a response rate of 18.4% may also limit our ability to generalize the findings due to non-responder bias. However, the sex distributions of the DMHS respondents (73.5% males) was representative of the overall DAN membership population (74.1% males), but the DMHS respondents were a little older (mean age 50.2 years) than the DAN membership (mean age 48.1 years; *p<0*.*001*) [4].

### Conclusions

DAN survey respondents were older than the general population and healthier, wealthier, white, with higher level of education, less likely to be unemployed, more physically active, fewer smokers, and more likely to get regular health check-ups. These better health indicators may signal a ‘healthy diver effect’ in an active diver population. On the other hand, DAN survey respondents were more likely to be heavy drinkers, which must be investigated further and intervened on. DAN survey respondents reported greater healthcare utilization than the general US population and the utilization of healthcare increased after 45 years of age, suggesting adherence to general fitness-to-dive recommendations among DAN recreational divers.

## Supporting information

S1 File DAN membership health survey questionnaire. (PDF)

S2 File De-identified DAN membership health survey data. (ZIP)
