# Supplementary material for: The healthy diver: A cross-sectional survey to evaluate the health status of recreational scuba diver members of Divers Alert Network (DAN)
Source: PLoS One. 2018 Mar 22;13(3):e0194380. doi: 10.1371/journal.pone.0194380 (PMC5864008; doi:10.1371/journal.pone.0194380)
Supplement: S1 File — (PDF) [file pone.0194380.s001.pdf]

Section 1: Dive Experience

1.1 **a.** Do you log your dives?

_____ All _____ Most _____ Few _____ None Radio buttons

b. How often do you dive? Radio buttons

- I take one dive trip a year and do not dive any other time
- I take several dive trips a year
- I dive at least once most months of the year

c. On an average, how many dives do you do every year?

_______________ Number box

1.2 How many dives have you made in total (lifetime)?

[ ] Number box

- number estimated
- number according to log book

1. 3 How many dives did you make during 2010? Number boxes

a. Standard Scuba air [ ] dives

b. Nitrox (Open-circuit) [ ] dives

c. Mixed gas/ Heliox/ Trimix [ ] dives

d. Rebreather (Closed-circuit) [ ] dives

1.4a Year of first diving certificate? [ ] Number box

1.4b What is your current level of diving certification? Radio buttons

_____ Basic
_____ Advanced
_____ Instructor

1.5 Year of open-circuit trimix certification?

Year: [ ] Number box

1.6 Year of closed-circuit rebreather (CCR) certification? Number boxes

Year: [ ] Number box

1.7 What type of dives have you practiced during the last one year? Radio buttons

a. Wreck	◯ Yes	◯ No
b. Penetrating wreck	◯ Yes	◯ No
c. Cave	◯ Yes	◯ No
d. Nitrox	◯ Yes	◯ No
e. Trimix	◯ Yes	◯ No
f. Rebreather	◯ Yes	◯ No
g. Ice diving	◯ Yes	◯ No
h. Dives deeper than 130 feet (40 meters)	◯ Yes	◯ No
i. Planned Decompression	◯ Yes	◯ No
j. Cold Water	◯ Yes	◯ No
k. Night	◯ Yes	◯ No
l. Low visibility	◯ Yes	◯ No
m. Underwater repair work	◯ Yes	◯ No
n. Altitude (> 999 feet above sea level)	◯ Yes	◯ No
o. High current	◯ Yes	◯ No

Section 2: Injuries

The following two pages deal with what you may have experienced concerning injuries and the causes of the injuries.

We define an injury as something you have experienced personally which needed medical treatment of any kind or caused disability that limits activities in your normal life.

On the following two pages we also ask you to report when the injuries occurred.
Before diving = from preparation before diving, (e.g., assembling equipment) until you descend under water.
During diving = While undertaking your dive, underwater.
After diving = from the point when you surface till you get home including disassembling, rinsing and taking care of the equipment.
Not related to diving = during performance of tasks other than diving-related activities and leisure time during all other times of the day than diving.

2.1 Have you personally had any type of injury during 2010 which needed medical treatment of any kind or disabled you in your normal life? Radio buttons

☐ YES ☐ NO

2.2.a Injuries and their relations to dive

Write the figure for the total number of injuries in column A and specify how it related to dive in columns C-F.

Number of times injured (A)	Type of injury (B)	Before Diving (C)	During Diving (D)	After Diving (E)	Not related to diving (F)
Number	Crushing injury and fractures	Check	Check	Check	Check
Number	Headache	Check	Check	Check	Check
Number	Overexertion, low back pain, sprain or strain	Check	Check	Check	Check
Number	Puncture, cut wound	Check	Check	Check	Check
Number	Sprain and strain of ankle	Check	Check	Check	Check
Number	Seasickness	Check	Check	Check	Check
Number	Hearing loss	Check	Check	Check	Check
Number	Ear problems (other than hearing loss)	Check	Check	Check	Check
Number	Sinus troubles	Check	Check	Check	Check
Number	Animal bites	Check	Check	Check	Check
Number	Burns	Check	Check	Check	Check
Number	Squeeze	Check	Check	Check	Check
Number	Allergic contact dermatitis	Check	Check	Check	Check
Number	Tooth pain	Check	Check	Check	Check
Number	Itching, after diving (<20 min)	Check	Check	Check	Check
Number	Loss of muscular strength (Paralysis)	Check	Check	Check	Check
Number	Pain in joints and/or muscles	Check	Check	Check	Check
Number	Unconsciousness	Check	Check	Check	Check
Number	Dizziness and giddiness	Check	Check	Check	Check
Number	Skin rash or marbling (several hours)	Check	Check	Check	Check
Number	Shortness of breath	Check	Check	Check	Check
Number	Other (please specify below)	Check	Check	Check	Check

2.3 Cause of injury

Match the causes of injuries to the injuries you indicated in the previous question. Select the appropriate cause you attribute your injury to and enter the corresponding letter in the box provided in front of the particular injury.

SINGLE TEXT BOX

EVENTS

Crushing Injury and fractures	Text
Headache	Text
Puncture or/and cut wound	Text
Sprain and strain of ankle	Text
Sprain and strain of wrist	Text
Seasickness	Text
Ear problems	Text
Dizziness and giddiness	Text
Sinus trouble	Text
Bite problems	Text
Frostbite	Text
Burn	Text
Itching after diving (<20 minutes)	Text
Skin rash or marbling (several hours)	Text
Limbs and Joints pain	Text
Squeeze	Text
Allergic contact dermatitis	Text
Hearing loss	Text
Tooth / Jaw pain	Text
Shortness of breath	Text
Other, what?	

Multiple Text box (maybe 50 letters)

____

____

____

Events that caused the injury

A Fall

B Overexertion injury, e.g. heavy lifting or cramp

C Mask or Suit squeeze

D Collision with an object

E Puncture or cut wound

F Exposure to heavy forces of nature (e.g. rough sea)

G Injured by person or animal

H Extreme cold

I Rapid ascent (more than 60 feet per minute)

J Traffic accident

K Accident with water vehicle (boat, underwater scooter, canoe)

L Exposure to electricity

M Too fast descent

N Out of air

O Decompression sickness

P Other, what?
………………………………
………………………………

2.4 During2010, did you claim any personal diving injury with an insurance company? Radio buttons

☐ YES ☐ NO

If yes, please give a short explanation: Pops up only if the above answer is yes

Text box ……………………………………………………………………………………
……………………………………………………………………………………………
……………………………………………………………………………………………
……………………………………………………………………………………………
……………………………………………………………………………………………
……………………………………………………………………………………………

2.5a Do you think that the injury you sustained could have been avoided?

_____Yes _____No Radio buttons

2.5b If yes, please describe how? Pops up only if the above answer is yes

Text box ……………………………………………………………………………………
……………………………………………………………………………………………
……………………………………………………………………………………………
……………………………………………………………………………………………
……………………………………………………………………………………………
……………………………………………………………………………………………

2.6 Do you have any suggestions of your own on how preventive measures can enhance diving safety?

Text box ……………………………………………………………………………………
……………………………………………………………………………………………
……………………………………………………………………………………………
……………………………………………………………………………………………
……………………………………………………………………………………………
……………………………………………………………………………………………
……………………………………………………………………………………………

2.7.a. During 2010, were you treated or advised to receive hyperbaric treatment?

Yes No Radio buttons

b. If yes, how many times? Pops up only if the above answer is yes
_ Number box_ times recommended ___ Number box__ times treated

2.8. a. During 2010, did you attempt 'In-water recompression' to treat symptoms of decompression sickness?

Yes No Radio buttons

b. If yes, how many times? Pops up only if the above answer is yes
__________ Number box

2.9. a. During 2010 did you receive oxygen first aid for dive related symptoms?

Yes No Radio buttons

b. If yes, how many times? Pops up only if the above answer is yes
_________ Number box

3. Underwater Environment:

Have you experienced the following hazards related to the underwater environment in 2010 while diving?

1. A. Entrapment or entanglement: e.g., seaweed, nets, fishing lines, etc.
   Yes No Radio buttons

   B. If yes, how many times has it occurred? Pops up only if the above answer is yes
   _________ Number box

2. A. Severe sea turbulence and undercurrents that caused difficulty in swimming or caused trouble carrying out tasks.
   Yes No Radio buttons

   B. If yes, how many times has it occurred? Pops up only if the above answer is yes
   ________ Number box

3. A. Injury due to attack by sea animals or sea plant poisoning.
   Yes No Radio buttons

   B. If yes, how many times has it occurred? Pops up only if the above answer is yes
   ________ Number box

4. A. Injury while working underwater on repairs of ships or other equipment.
   Yes No Radio buttons

   B. If yes, how many times has it occurred? Pops up only if the above answer is yes
   ________ Number box

5. A. Injury due to underwater explosion / electric shock.

Yes No Radio buttons

B. If yes, how many times has it occurred? Pops up only if the above answer is yes
________ Number box

4. Dive safety and diving rules:
Issues with diving equipment:

1. A. How much gas do you keep for your ascent?
_______ Number box psi bars Radio buttons

B. Did you ever run out of breathing gas while underwater?

Yes No Radio buttons

C. If yes, how many times did it happen during 2010? Pops up only if the above answer is yes

_________ Number box

2. A. Do you dive with a buddy?

____ Never ____Sometimes ____Always Radio buttons

B. Do you ever get separated from your buddy?
_____ Never ____Sometimes _____Often Radio buttons

3. A. What is your usual ascent rate?
____ Number box f/min m/min Radio buttons

B. How many times in 2010 did you have to do an emergency ascent?
____________________ Number box

C. What kind of emergency? Please explain.

____ Text box

D. How many times in 2010 did you have to do a free ascent (i.e., without the regulator in your mouth)?
____________________ Number box

4. A. Have you had any problems because of mask squeeze?

Yes No Radio buttons

B. Have you had problems equalizing that have prevented you from diving?

Yes No Radio buttons

5. A. Do you own a buoyancy compensator device (BCD)?

Yes No Radio buttons

B. Did you ever have your buoyancy compensator device (BCD) malfunction?

Yes No Radio buttons

5. **Unreported injuries:**

How many times in 2010 did you experience any of the following injuries (as a result of diving) for which you did NOT seek medical treatment and did NOT report? Radio buttons

Corresponding number box must get activated only when the Yes is answered

1. Joint pains / Muscle pains / limb pain	Yes	No	__Number box___	times
2. Skin rash / Marbled skin	Yes	No	__Number box___	times
3. Fullness, pain or bleeding from ear	Yes	No	__Number box___	times
4. Sinusitis / Headache	Yes	No	__Number box___	times
5. Cuts, scrapes and wounds	Yes	No	__Number box___	times
6. Cuts, scrapes and wound infections	Yes	No	__Number box___	times
7. Dizziness/ Vertigo not related to seasickness	Yes	No	__Number box___	times
8. Seasickness	Yes	No	__Number box___	times
9. Temporary hearing loss	Yes	No	__Number box___	times
10. Tooth pain	Yes	No	__Number box___	times
11. Numbness and tingling in limbs	Yes	No	__Number box___	times
12. Muscle weakness	Yes	No	__Number box___	times
13. Extreme fatigue	Yes	No	__Number box___	times
14. Confusion and Disorientation	Yes	No	__Number box___	times
15. Others	Yes	No	__Number box___	times

If others, please specify ________Text box___________________________________

Section 6: Health Status

6.1 Would you say that in general your health is—

Radio buttons

Please check one answer.

- Excellent
- Very Good

- Good
- Fair
- Poor
- Don't know / Not sure

Section 7: Healthy Days — Health-Related Quality of Life

7.1 Now thinking about your physical health, which includes physical illness and injury, for how many days during the past 30 days was your physical health not good?

______ Number of days

- None
- Don't know / Not sure

7.2 Now thinking about your mental health, which includes stress, depression, and problems with emotions, for how many days during the past 30 days was your mental health not good?

______ Number of days

- None
- Don't know / Not sure

7.3 During the past 30 days, for about how many days did poor physical or mental health keep you from doing your usual activities, such as self-care, work, or recreation?

______ Number of days

- None
- Don't know / Not sure

Section 8: Health Care Access

8.1 Do you have one person you think of as your personal doctor or health care provider?

- Yes, only one
- More than one
- No
- Don’t know / Not sure

8.2 About how long has it been since you last visited a doctor for a routine checkup? A routine checkup is a general physical exam, not an exam for a specific injury, illness, or condition.

- Within past year (anytime less than 12 months ago)
- Within past 2 years (1 year but less than 2 years ago)
- Within past 5 years (2 years but less than 5 years ago)
- 5 or more years ago
- Don’t know / Not sure
- Never

Section 9: Exercise & Physical Activity

9.1 During the past month, other than your regular job, did you participate in any physical activities or exercises such as running, calisthenics, golf, gardening, or walking for exercise?

- Yes
- No
- Don’t know / Not sure

9.2 When you are at work, which of the following best describes what you do? Would you say—

If you have multiple jobs, include all jobs.

- Mostly sitting or standing
- Mostly walking
- Mostly heavy labor or physically demanding work
- Don’t know / Not sure

We are interested in two types of physical activity - vigorous and moderate. Vigorous activities cause large increases in breathing or heart rate while moderate activities cause small increases in breathing or heart rate.

9.3 Now, thinking about the moderate activities you do when you are not working (employed or self-employed) in a usual week, do you do moderate activities for at least 10 minutes at a time, such as brisk walking, bicycling, vacuuming, gardening, or anything else that causes some increase in breathing or heart rate?

- ☐ Yes
- ☒ No [Go to Q12.6]
- ☐ Don't know / Not sure [Go to Q9.6]

9.4 How many days per week do you do these moderate activities for at least 10 minutes at a time?

______ Days per week

- ☐ Do not do any moderate physical activity for at least 10 minutes at a time [Go to Q9.6]
- ☐ Don't know / Not sure [Go to Q9.6]

9.5 On days when you do moderate activities for at least 10 minutes at a time, how much total time per day do you spend doing these activities?

______ Hours and minutes per day

- ☐ Don't know / Not sure

9.6 Now, thinking about the vigorous activities you do ["when you are not working" if employed or self-employed] in a usual week, do you do vigorous activities for at least 10 minutes at a time, such as running, aerobics, heavy yard work, or anything else that causes large increases in breathing or heart rate?

- ☐ Yes
- ☐ No [Go to next section]
- ☐ Don't know / Not sure [Go to next section]

9.7 How many days per week do you do these vigorous activities for at least 10 minutes at a time?

______ Days per week

- ☐ Do not do any vigorous physical activity for at least 10 minutes at a time [Go to next section]
- ☐ Don't know / Not sure [Go to next section]

9.8 On days when you do vigorous activities for at least 10 minutes at a time, how much total time per day do you spend doing these activities?

________ Hours and minutes per day

☐ Don't know / Not sure

Section 10: Diabetes

10.1 Have you been tested for high blood sugar or diabetes within the past three years?

☐ Yes

☐ No

☐ Don't know / Not sure

10.2 Have you ever been told by a doctor that you have diabetes?

☐ Yes

FEMALES: If "Yes, was this only when you were pregnant?

☐ Yes, (female) told only during pregnancy

☐ No

☐ No, pre-diabetes or borderline diabetes

☐ Don't know / Not sure

10.3 Are you now taking insulin?

☐ Yes

☐ No

10.4. Are you taking any oral anti-diabetic medications?

_____ Yes
_____ No

Section 11: Cardiovascular Disease Prevalence

Has a doctor, nurse, or other health professional EVER told you that you had any of the following? For each, check "Yes," "No," or "Not sure."

11.1 (Ever told) you had a heart attack, also called a myocardial infarction?

☐ Yes

☐ No

☐ Don't know / Not sure

11.2 (Ever told) you had angina or coronary heart disease?

- ☐ Yes
- ☐ No
- ☐ Don't know / Not sure

11.3 (Ever told) you had a stroke?

- ☐ Yes
- ☐ No
- ☐ Don't know / Not sure

11.4 Do you take any medication for heart disease?

- ☐ None
- ☐ Antiarhhythmic drugs
- ☐ Other Name of other drugs: ______________________________

11.5 Have you ever had heart surgery?

- ☐ Yes

Describe: ______________________________

- ☐ No

11.6 Do you have a pacemaker?

- ☐ Yes
- ☐ No

Section 11A: Cholesterol

11A.1 Have you ever been told that you have high blood cholesterol?

- ☐ Yes
- ☐ No
- ☐ Don't know / Not sure

11A.2 When was the last time you checked your blood cholesterol?

- Never [skip Q11.A.3 & Q11.A.4]
- <1 year
- >1 year

11A.3 What was the total cholesterol (TC) in mg/dl?

- <200
- 200-239
- >240
- not sure/don't know

11A.4 What was your high density level cholesterol HDL-C in mg/dl?

- <35
- 35-59
- >60
- not sure/don't know

Section 11B: Blood Pressure

11B.1 Have you ever been told that you have high blood pressure?

- Yes
- No
- Don’t know / Not sure

11B.2 When was the last time you measured your blood pressure?

- <6 months ago
- 6-12 months ago
- >1 year ago
- Never [Skip Q11.B.3]

11B.3 What were the most recent measurements of your blood pressure?

Systolic	Diastolic
☐ <130	☐ <85
☐ 130-139	☐ 85-89
☐ 140-159	☐ 90-99
☐ >160	☐ >100

Section 12: Asthma

12.1 Have you ever been told by a doctor, nurse, or other health professional that you had asthma?

- ☐ Yes
- ☐ No [Go to next section]
- ☐ Don't know / Not sure [Go to next section]

12.2 Do you still have asthma?

- ☐ Yes
- ☐ No
- ☐ Don't know / Not sure

Section 13: Disability

The following questions are about health problems or impairments you may have.

13.1 Are you limited in any way in any activities because of physical, mental, or emotional problems?

- ☐ Yes
- ☐ No
- ☐ Don't know / Not sure

13.2 Do you now have any health problem that requires you to use special equipment, such as a cane, a wheelchair, a special bed, or a special telephone?

Include occasional use or use in certain circumstances.

- ☐ Yes
- ☐ No
- ☐ Don't know / Not sure

Section 14: Tobacco Use

14.1 Have you smoked at least 100 cigarettes in your entire life?

NOTE: 5 packs = 100 cigarettes

- Yes
- No [Go to next section]
- Don't know / Not sure [Go to next section]

14.2 Do you now smoke cigarettes every day, some days, or not at all?

- Every day
- Some days
- Not at all [Go to next section]
- Don't know / Not sure [Go to next section]

14.3 During the past 12 months, have you stopped smoking for one day or longer because you were trying to quit smoking?

- Yes
- No
- Don't know / Not sure

Section 15: Demographics

15.1 Sex?

- Male
- Female

15.2 What is your age?

________ years

15.3 Are you Hispanic or Latino?

- Yes
- No
- Don't know / Not sure

15.4 Which one or more of the following would you say is your race?

(Check all that apply) Check boxes

- ☐ White
- ☐ Black or African American
- ☐ Asian
- ☐ Native Hawaiian or Other Pacific Islander
- ☐ American Indian or Alaska Native
- ☐ Other [specify] ____________________
- ☐ Don't know / Not sure

15.5 What is the highest grade or year of school you completed?

- ☐ Never attended school or only attended kindergarten
- ☐ Grades 1 through 8 (Elementary)
- ☐ Grades 9 through 11 (Some high school)
- ☐ Grade 12 or GED (High school graduate)
- ☐ College 1 year to 3 years (Some college or technical school)
- ☐ College 4 years or more (College graduate)

15.6 Are you currently…?

- ☐ Employed for wages
- ☐ Self-employed
- ☐ Out of work for more than 1 year
- ☐ Out of work for less than 1 year
- ☐ A Homemaker
- ☐ A Student
- ☐ Retired
- ☐ Unable to work

15.7 Is your annual household income from all sources:

- ☐ Less than $25,000

- ☐ Less than $50,000 ($25,000 to $49,000)
- ☐ Less than $100,000 ($50,000 to $100,000)
- ☐ Less than 150,000 ($100,000 to 149,000)
- ☐ More than $150,000
- ☐ Don’t know / Not sure

15.8 About how much do you weigh without shoes?

[ ] ☐ pounds ☐ kilograms

☐ Don't know / Not sure

15.9 About how tall are you without shoes?

[ ] ☐ ft/inches ☐ meters/centimeters

☐ Don't know / Not sure

15.10 **How much did you weigh a year ago?** [FEMALES: If you were pregnant a year ago, how much did you weigh before your pregnancy?]

[ ] ☐ pounds ☐ kilograms

☐ Don't know / Not sure

Section 16: Alcohol Consumption

16.1 During the past 30 days, have you had at least one drink of any alcoholic beverage such as beer, wine, a malt beverage or liquor?

- ☐ Yes
- ☐ No [Go to next section]
- ☐ Don't know / Not sure [Go to next section]

16.2 During the past 30 days, how many days per week or per month did you have at least one drink of any alcoholic beverage?

- ______ Days per week
- ______ Days in past 30 days
- ☐ No drinks in past 30 days [Go to next section]
- ☐ Don't know / Not sure

16.3 One drink is equivalent to a 12-ounce beer, a 5-ounce glass of wine, or a drink with one shot of liquor. During the past 30 days, on the days when you drank, about how many drinks did you drink on the average?

NOTE: A 40 ounce beer would count as 3 drinks, or a cocktail drink with 2 shots would count as 2 drinks.

- ______ Number of drinks
- ☐ None
- ☐ Don't know / Not sure

16.4 Considering all types of alcoholic beverages, how many times during the past 30 days did you have one or more drinks on an occasion?

- ______ Number of times
- ☐ None
- ☐ Don't know / Not sure

16.5 During the past 30 days, what is the largest number of drinks you had on any occasion?

- ______ Number of drinks
- ☐ Don't know / Not sure

Section 17: Emotional Support and Life Satisfaction

17. In general, how satisfied are you with your life?

- ☐ Very satisfied
- ☐ Satisfied
- ☐ Dissatisfied
- ☐ Very dissatisfied
- ☐ Don't know / Not sure
